# Combined Application of Probiotic and Phytobiotic Additives Improves Fermentation Quality and Amino Acid Preservation in Tropical Forage Silages

**DOI:** 10.3390/ani16142224

**Published:** 2026-07-17

**Authors:** Irwan Susanto, Komang G. Wiryawan, Anuraga Jayanegara, Farisha R. Azzahra, Roni Ridwan, Rohmatussolihat, Alwan Farhandhany, Mardiah Rahmadani, Erika B. Laconi

**Affiliations:** 1 Department of Nutrition and Feed Technology, Faculty of Animal Science, IPB University, Bogor 16680, Indonesia; irwansusanto939@gmail.com (I.S.); anuraga.jayanegara@gmail.com (A.J.); frazzahra02@gmail.com (F.R.A.); mardiahrahmadani123@gmail.com (M.R.); erika_laconi@apps.ipb.ac.id (E.B.L.); 2 Feed Technology Division, PT. Central Proteina Prima, Kuningan, Jakarta 12920, Indonesia; 3 Animal Feed and Nutrition Modelling Research Group (AFENUE), IPB University, Bogor 16680, Indonesia; 4 Research Center for Applied Zoology, National Research and Innovation Agency, Indonesia, BRIN, Bogor 16911, Indonesia; roni001@brin.go.id (R.R.); rohmatussolihat@gmail.com (R.); 5 Bioresources and Life Sciences, United Graduate School of Agricultural Sciences, Tottori University, Tottori 680-0945, Japan; afarhandany@gmail.com

**Keywords:** amino acid, deamination, phytobiotics, probiotics, silage

## Abstract

Preserving high-quality forage through silage is essential for continuous livestock production, yet protein degradation during fermentation remains a major challenge. To mitigate this nutrient loss, probiotics and phytobiotics are utilized as protective additives. This study investigated the synergistic application of lactic acid bacteria (LAB) and plant-derived bioactive compounds as deamination inhibitors to improve the fermentation quality and amino acid profiles of tropical forage silages. The experiment followed a 2 × 4 × 4 completely randomized factorial design with five replications. Factor A tested the inclusion of lactic acid bacteria (LAB) inoculum (*Lactiplantibacillus plantarum*). Factor B compared different plant extracts as additives (*Acacia mangium*, *Swietenia macrophylla*, and cumin essential oil), while Factor C evaluated various forage types (*Pennisetum purpureum*, *Indigofera zollingeriana*, *Gliricidia sepium*, and *Stylosanthes guianensis*). Statistical analysis revealed that LAB inoculation significantly reduced the pH level, suppressed fungal contamination, and improved physical traits (*p* < 0.05). Both the additive sources and forage types also exerted strong influences across all measured variables (*p* < 0.05). The interaction between microbes and plant extracts enhances protein preservation during ensiling. Among all the treatments tested, *Indigofera zollingeriana* showed the best response. The specific combination of *L. plantarum* and *A. mangium* extracts in *Indigofera* silage produced the best fermentation characteristics and a high ability to retain essential amino acids, particularly histidine, lysine, tryptophan, and valine. Lower NH_3_-N concentrations and better amino acid preservation in this treatment indicate reduced protein loss due to proteolysis and deamination during fermentation. These findings confirm that the combination of probiotics and certain plant extracts is a highly effective strategy for maintaining the nutritional integrity of tropical forage preserved in silage form.

## Simple Summary

Protein degradation during ensiling reduces forage nutritional value, particularly through the loss of essential amino acids. This study evaluated whether combining the probiotic *Lactiplantibacillus plantarum* with phytobiotic extracts from *Acacia mangium*, *Swietenia macrophylla*, and cumin essential oil could improve silage quality and preserve nutrients in four tropical forages. LAB inoculation enhanced fermentation by lowering pH and improving the physical quality of silage, while phytobiotic extracts helped preserve nutrient composition. Among the tested forages, *Indigofera zollingeriana* produced the highest-quality silage. The combination of *L. plantarum* and *A. mangium* extracts was the most effective treatment for maintaining essential amino acids, including histidine, lysine, tryptophan, and valine. These findings demonstrate that combining probiotics and phytobiotics is a practical approach to improving silage quality, preserving protein value, and supporting more sustainable ruminant production.

## 1. Introduction

Silage production relies on lactic acid bacteria (LAB) to preserve forage through controlled fermentation. During this process, a diverse community of microorganisms generates various metabolites that fundamentally dictate storage success, feed utilization, and subsequent livestock productivity [1]. As a cornerstone of modern farming systems, this preservation method secures a steady supply of high-quality feed, bridging the gap during seasonal shortages. Beyond ensuring year-round availability, the technology drastically reduces nutrient losses that typically occur through feed spoilage and degradation, resulting in better overall feed efficiency.

Evaluating silage quality depends heavily on its storage stability and nutrient profile, particularly the levels of crude protein and crude fiber. When ensiling high-protein forages like *Indigofera*, *Gliricidia*, and *Stylosanthes*, intensive proteolysis often occurs. This protein breakdown and subsequent deamination generate ammonia nitrogen (NH_3_-N) and free amino acids [2]. Consequently, the concentration of accumulated NH_3_-N serves as a primary benchmark for quality, where elevated levels directly signal severe protein degradation [3]. This degradation typically stems from opportunistic microbial activity, such as *Clostridia* proliferation, which compromises the efficiency of protein utilization in livestock.

During the fermentation of organic matter, amino acid deamination is a major pathway of nitrogen metabolism, converting amino acids into ammonia nitrogen (NH_3_-N) and carbon skeletons. In silage, excessive deamination is commonly associated with the proliferation of undesirable anaerobic microorganisms under unfavorable ensiling conditions, including high moisture content, low dry matter concentration, high buffering capacity, and inadequate acidification (pH > 4.5) [4]. These conditions accelerate proteolysis and increase NH_3_-N accumulation, leading to nitrogen losses and reduced nutritional value. Therefore, minimizing deamination and preserving true protein during ensiling are important objectives for improving silage quality.

To optimize silage quality, researchers and producers frequently incorporate feed additives such as LAB probiotics, organic acids, and plant-derived phytobiotics. These additives direct the fermentation process, suppress aerobic spoilage, and preserve essential nutrients during storage [5,6]. Among these approaches, inoculation with *Lactiplantibacillus plantarum* is one of the most widely adopted strategies for improving silage fermentation [7]. This LAB strain rapidly produces lactic acid, resulting in a rapid decline in pH that inhibits undesirable microbial activity and minimizes nutrient losses during ensiling. A study by Rohmatussolihat et al. [8] demonstrated that inoculation with different LAB strains at a dose of 1 × 10^6^ CFU g^−1^ fresh material (equivalent to 2.5 × 10^5^ CFU g^−1^ for each strain) significantly reduced silage pH and improved nutrient preservation throughout the storage period. This rapid acidification suppresses undesirable enzymatic activity and inhibits fermentative degradation, thereby preserving the nutritional value of the forage.

Plant-derived bioactive components, specifically phytochemicals such as tannins, saponins, and essential oils, serve as natural silage additives. Tannins possess a high protein-binding capacity, which suppresses NH_3_-N production and reduces amino acid deamination during ensiling [2,9]. Beyond protecting proteins, these phenolic compounds disrupt the metabolic pathways and proliferation of proteolytic microorganisms [10]. Saponins complement this activity by exerting antimicrobial effects that modulate the fermentation pathway [11]. Additionally, the antimicrobial and antioxidant properties inherent in essential oils stabilize the silage environment, further minimizing NH_3_-N accumulation [12]. Utilizing these diverse plant metabolites provides a mechanism to improve feed efficiency and livestock production outcomes. The inclusion level of phytobiotics evaluated in this study was determined based on a preliminary meta-regression analysis [13], which identified 9–10 g/kg (approximately equivalent to 1% inclusion) as the optimal concentration for minimizing NH_3_-N production. This relationship was defined by the regression equation: NH_3_-N = −2.105 + (−0.003 × inclusion level).

The phytobiotic additives used in the present study were derived from three plant sources with distinct phytochemical profiles, namely *Acacia mangium* bark, Swietenia macrophylla bark, and cumin (*Cuminum cyminum*) essential oil. *Acacia mangium* bark was selected because it is rich in condensed tannins, which readily form tannin–protein complexes that protect proteins from excessive proteolysis, thereby improving nitrogen retention during the ensiling process [14]. In contrast, *Swietenia macrophylla* bark contains abundant phenolic compounds and flavonoids with potent antimicrobial and antioxidant properties that can suppress undesirable microorganisms while minimizing oxidative degradation of nutrients [15]. Meanwhile, cumin essential oil is characterized by high concentrations of bioactive constituents, particularly cuminaldehyde and various terpenoids, which exhibit broad-spectrum antimicrobial activity capable of modulating silage fermentation through the inhibition of spoilage microorganisms [16].

Recent molecular docking and molecular dynamics studies demonstrated that bioactive compounds from tannin, saponin, and cumin-derived phytochemical groups exhibit high binding affinity toward *glutamate dehydrogenase*, indicating their potential to inhibit enzymatic deamination during the ensiling process [17,18]. Due to their distinct phytochemical profiles and mechanisms of action, these three phytobiotic sources provide a suitable model to evaluate how different classes of plant bioactive compounds influence fermentation characteristics, nitrogen metabolism, and amino acid preservation during the ensiling of tropical forage legumes. Furthermore, these phytochemicals may operate in combination with LAB to suppress undesirable microbial populations while protecting proteins and free amino acids from extensive degradation, thereby improving silage quality and nitrogen conservation. Therefore, the present study evaluated these bioinformatics-based predictions under practical ensiling conditions by determining amino acid profiles and NH_3_-N concentrations as indicators of deamination. The preservation of specific amino acids despite altered NH_3_-N levels indicates that the combined phytobiotic–LAB application modulates deamination pathways to enhance amino acid retention, supporting the hypothesis generated from the molecular simulations.

To explore these mechanisms further, this study evaluated the effects of *Acacia mangium* bark extract, *Swietenia macrophylla* bark extract, and cumin essential oil applied at 10 g/kg of fresh forage, either in the presence or absence of LAB inoculation, across different tropical forages. We hypothesized that the addition of these phytochemical compounds would improve silage fermentation by suppressing proteolytic microorganisms, binding specific amino acids, and reducing amino acid deamination, thereby lowering NH_3_-N accumulation and enhancing nutrient preservation. Therefore, the objective of this study was to evaluate the distinct impacts of various phytochemical additives, both alone and in combination with LAB inoculation, on the preservation characteristics and nutritional quality of high-protein silage.

## 2. Materials and Methods

### 2.1. Research Location

This study was conducted across multiple facilities, including the Integrated Laboratory, the Meat and Working Animal Nutrition Laboratory, and the Feed Science and Technology Laboratory within the Department of Nutrition Science and Feed Technology, Faculty of Animal Science, IPB University. Additionally, sample analyses and experimental procedures were carried out at the Feed Bioprocess Research Group Laboratory, Research Center for Zoology, National Research and Innovation Agency (BRIN) in Cibinong, Bogor.

### 2.2. Sample Preparation and Extraction

Bark samples of *Acacia mangium* and *Swietenia macrophylla* (mahogany) were harvested from the forest area of the IPB University campus in Bogor, while black cumin essential oil was obtained from a commercial supplier. The collected bark samples were oven-dried at 60 °C for 48 h, pulverized, and passed through an 80-mesh sieve to produce a fine crude powder. The extraction procedure followed a modified protocol based on Makkiyah et al. [19] utilizing the Microwave-Assisted Extraction (MAE) method. Briefly, 4 g of each bark powder was suspended in 40 mL of analytical-grade (pro-analysis) methanol to achieve a 1:10 (m/v) ratio in an Erlenmeyer flask. This suspension was then irradiated in a microwave oven (Sharp R-21D0(S)-IN, Sharp Corporation, Osaka, Japan) at 135 W for 3 min. After irradiation, the extract was cooled to room temperature and filtered under vacuum to obtain the crude methanolic extract solution. The extraction yield was 16% for *A. mangium* bark and 12% for *S. macrophylla* bark (*w*/*w*, based on dry bark weight). The resulting filtrate was concentrated using a rotary evaporator at 40 °C under reduced pressure until the methanol was completely removed. The concentrated extract was then reconstituted with sterile distilled water to obtain a standardized extract concentration equivalent to 0.1 g crude bark/mL before its application to the silage treatments. This procedure ensured that no residual methanol was introduced into the experimental silages.

### 2.3. Total Phenolic Content (TPC) Analysis

The total phenolic content of the extracts was quantified via the Folin–Ciocalteu method, adapted with modifications from Makkiyah et al. [19], and expressed as gallic acid equivalents (GAE). A standard calibration curve was initially established using gallic acid concentrations ranging from 0 to 300 ppm. For the assay, a 20 µL aliquot of either the plant extract or the gallic acid standard was dispensed into a 96-well microplate and blended with 120 µL of 10% Folin–Ciocalteu reagent. Following a 5 min incubation in the dark, 80 µL of 10% Na_2_CO_3_ was added to the mixture. The reaction was then incubated in the dark for an additional 30 min. Finally, absorbance changes were recorded at 750 nm utilizing a microplate spectrophotometer (SPECTROstar Nano, BMG LABTECH GmbH, Ortenberg, Germany).

### 2.4. Total Flavonoid Content (TFC) Analysis

The total flavonoid content was quantified utilizing the aluminum chloride (AlCl_3_) colorimetric assay and expressed as quercetin equivalents (QE), following a modified protocol from Khumaida et al. [20]. The reaction was performed within a 96-well microplate by introducing a 10 µL aliquot of either the plant extract or the quercetin standard, which was subsequently diluted with 60 µL of methanol. To this mixture, 10 µL of 10% AlCl_3_, 10 µL of 1 M acetic acid solution, and 120 µL of distilled water were sequentially added. After a 30 min incubation period at room temperature, the optical density was recorded at 415 nm employing a microplate spectrophotometer (SPECTROstar Nano, BMG LABTECH).

### 2.5. Total Tannin Content (TTC) Analysis

The total tannin content was quantified via the Folin–Ciocalteu method, adapted with modifications from Islam et al. [21], and expressed as tannic acid equivalents (TAE). Standard calibration solutions were prepared using tannic acid across a six-point concentration gradient (6.25, 12.5, 25, 50, 100, and 200 µg/mL). For the analysis, a 0.1 mL aliquot of the sample filtrate (200 µg/mL) was transferred into a test tube containing 7.5 mL of distilled water. Subsequently, 0.5 mL of Folin–Ciocalteu phenol reagent and 1 mL of 35% sodium carbonate (Na_2_CO_3_) solution were introduced, and the final volume was brought up to 10 mL using distilled water. After being thoroughly vortexed, the reaction mixture was incubated at room temperature for 30 min. The resulting absorbance was recorded against a reagent blank at 725 nm employing a UV-Vis spectrophotometer (Shimadzu UV PC-1600, Shimadzu Corporation, Kyoto, Japan).

### 2.6. Antioxidant Capacity: 2,2-Diphenyl-1-Picrylhydrazyl (DPPH) Assay

The radical scavenging activity of the cumin essential oil, acacia, and mahogany bark samples was evaluated using the DPPH assay and expressed as Trolox equivalents (TE) per gram of dry weight (µmol TE g^−1^ DM), adapted from Nurcholis et al. and Aryal et al. [22,23]. For reagent preparation, a 1.25 µM DPPH radical solution was synthesized by dissolving 2.5 mg of DPPH powder in analytical-grade ethanol to a final volume of 50 mL. Separately, a 1000 ppm stock solution was prepared by dissolving 0.0025 g of Trolox in 100 mL of pro-analysis ethanol, which was subsequently diluted to establish a standard calibration curve ranging from 20 to 90 µM. The assay was performed by dispensing a 100 µL aliquot of either the sample or the Trolox standard into a 96-well microplate (Biologix Group Limited, Jinan, China), followed by the addition of 100 µL of the DPPH working solution. After a 30 min incubation period in the dark, the resulting optical density was recorded at 515 nm utilizing a microplate spectrophotometer (BMG LABTECH, GmbH, Ortenberg, Germany).

### 2.7. Silage Preparation

The forage materials evaluated in this study comprised Napier grass (*Pennisetum purpureum*), *Indigofera zollingeriana*, *Gliricidia sepium*, and *Stylosanthes guianensis*, all harvested from the Agrostology Grassland at the Faculty of Animal Science, IPB University. For silage production, only the edible portions of the plants—specifically the leaves and tender stems—were selected. The baseline nutritional profiles of these raw materials are compiled in Table 1. Prior to processing, the harvested forages were air-dried to adjust their moisture content before weighing. The plant materials were then chopped into uniform segments of approximately 2–3 cm. For each experimental unit, a 200 g aliquot of chopped forage was weighed and thoroughly mixed with the respective plant extract treatment at an inclusion level of 1% (*w*/*w*) based on the fresh silage weight, according to the predefined experimental design. This inclusion level was selected based on the findings of a previous meta-regression study, which identified 1% as the optimal dose for decreasing NH_3_-N production [13]. The same inclusion level was applied across all treatments to standardize the experimental conditions and to evaluate the practical feasibility of using these plant extracts as silage additives under realistic farming conditions.

For the probiotic treatments, an *L. plantarum* inoculum (containing 1 × 10^8^ CFU mL^−1^) was introduced at a concentration of 0.2 mL per 200 g of fresh forage. The treated materials were immediately transferred into 300 mL plastic bags, vacuum-sealed to establish strict anaerobic conditions, and ensiled at room temperature for 30 days. Following the incubation period, the solid and liquid fractions were separated for downstream analysis. To prepare the liquid extract, a 10 g aliquot of fresh silage was homogenized with 90 mL of distilled water and filtered. The remaining solid mass was oven-dried at 60 °C for 48 h and pulverized into a fine powder. The dried biomass was subsequently utilized for nutritional composition analysis, while the aqueous filtrate was reserved to evaluate fermentative characteristics.

### 2.8. Organoleptic and Physical Quality Analysis

Upon opening the plastic bags, the sensory profiles of the silage were immediately evaluated based on color, aroma, texture, and the incidence of visible mold contamination. The scoring system for this organoleptic assessment followed the standardized protocols described by Zakariah et al. [24], as detailed in Table 2. Each physical attribute was quantitatively graded using a structured scoring scale ranging from 1 (excellent) to 4 (poor). The physical quality of silage was evaluated by five trained panelists experienced in silage quality assessment. Prior to the evaluation, the panelists were familiarized with the scoring criteria. Samples were coded with random numbers and presented in a randomized order to minimize bias. Physical characteristics, including color, odor or aroma, texture, and visible mold, were scored according to the established evaluation criteria.

### 2.9. Nutrient and Amino Acid Composition Analysis

The nutritional and amino acid profiles of the ensiled biomass were determined via Near-Infrared Reflectance Spectroscopy (NIRS), adapting the methodology described by Parastiwi et al. [25]. Spectral measurements were acquired utilizing a Buchi NIRFlex N500 Fourier Transform Near-Infrared (FT-NIR) spectrometer (BÜCHI Labortechnik AG, Flawil, Switzerland). For each scan, a 25 g aliquot of the dried, pulverized silage was uniformly distributed into a Petri dish and positioned onto a rotating sample holder. The samples were scanned across a near-infrared wavelength spectrum ranging from 1000 to 2500 nm, under a controlled ambient temperature of 16–20 °C. To ensure reproducibility and minimize experimental bias, all spectral analyses were performed in duplicate.

Amino acid profiles and chemical composition were determined using Fourier Transform Near-Infrared Spectroscopy (FT-NIRS) employing an established calibration model developed for tropical forage samples. The calibration model was previously developed and verified against conventional wet chemistry reference analyses using an extensive tropical forage database, as described by Parastiwi et al. [25]. The validation performance of the calibration model used in this study, including the calibration range, Standard Error of Prediction (SEP), bias, and slope, is presented in Supplementary Table S1.

### 2.10. pH Measurement

The pH of the ensiled material was determined directly from the prepared aqueous silage extract. All potentiometric measurements were conducted using a digital pH meter (Lutron, model pH-208, Lutron Electronic Enterprise Co., Ltd., Taipei, Taiwan) at room temperature (25 ± 2 °C). Prior to sample analysis, the electrode was calibrated and standardized using fresh commercial buffer solutions at pH 4.0 and 7.0 to ensure instrument accuracy.

### 2.11. Lactic Acid Measurement

Lactic acid concentration was quantified spectrophotometrically based on the colorimetric method described by Borshchevskaya et al. [26]. Briefly, a 50 µL aliquot of the aqueous silage extract was transferred into a test tube and reacted with 2 mL of a 0.2% ferric chloride (FeCl_3_) solution. The mixture was immediately vortexed for 15 s to ensure complete homogenization. Finally, the optical density of the resulting complex was recorded at a wavelength of 390 nm employing a UV-Vis spectrophotometer (Shimadzu UV PC-1600, Shimadzu Corporation, Kyoto, Japan).

### 2.12. Ammonia Nitrogen (NH_3_-N) Concentration Measurement

The concentration of ammonia nitrogen (NH_3_-N) was quantified using the method adapted from Souza et al. [27]. For the assay, a 100 µL aliquot of the aqueous silage extract was dispensed into a test tube, followed by the sequential addition of 1.5 mL of phenol solution and 1.5 mL of sodium hypochlorite (NaOCl) solution. The reaction mixture was thoroughly mixed and subsequently incubated in a water bath maintained at 39 °C for 15 min. Finally, the resulting absorbance was recorded at a wavelength of 630 nm utilizing a UV-Vis spectrophotometer.

### 2.13. Design, Parameters, and Data Analysis

The experiment was arranged in a 2 × 4 × 4 completely randomized factorial design (CRD) with five replications per treatment combination and total 160 bag silage. The three experimental factors were defined as follows:

**Factor A (LAB Inoculation):** Consisted of two levels, comprising a control group (without inoculum) and a probiotic group inoculated with *L. plantarum*.

**Factor B (Phytochemical Additives):** Consisted of four treatments, including a negative control (P0, no additive), *Acacia mangium* bark extract (P1), *Swietenia macrophylla* bark extract (P2), and black cumin essential oil (P3). All additives were incorporated at a standardized dose of 10 g/kg as feed.

**Factor C (Forage Types):** Comprised four distinct forage species, namely Napier grass (*Pennisetum purpureum*, R1), *Indigofera zollingeriana* (R2), *Gliricidia sepium* (R3), and *Stylosanthes guianensis* (R4).

The collected data were analyzed using analysis of variance (ANOVA). Prior to analysis, data normality was assessed using the Shapiro–Wilk test, and data transformation was performed when necessary to satisfy the assumptions of ANOVA. When significant treatment effects were detected, means were compared *using Duncan’s Multiple Range Test* (DMRT) at a significance level of *p* < 0.05 [28]. Data curation, assumption testing, data transformation, and statistical analyses were performed using Microsoft Excel and IBM SPSS Statistics version 25.0 (IBM Corp., Armonk, NY, USA).

Phytochemical content and antioxidant capacity data were further evaluated using Pearson’s correlation analysis following the method of Batubara et al. [29]. The correlation analysis was performed using the *PerformanceAnalytics* package in RStudio version 4.2.2 (R Foundation for Statistical Computing, Vienna, Austria). All independent observations from the phytochemical analyses were included in the correlation analysis. Each phytobiotic source was represented by four independent biological replicates, with each biological replicate considered an independent experimental unit, resulting in a total of 12 independent observations (*n* = 12) for each parameter included in the correlation analysis. The main evaluated variables encompassed four primary categories:

1. *Sensory and physical traits*, including color, aroma, texture, and visible mold contamination.
2. *Fermentation characteristics*, monitoring pH dynamics, lactic acid concentrations, and ammonia accumulation.
3. *Silage nutrient content*, quantifying crude protein, ether extract, crude fiber, total ash, neutral detergent fiber (NDF), acid detergent fiber (ADF), calcium (Ca), and phosphorus (P).
4. *Amino acid profiles*, mapping both essential and non-essential amino acid concentrations.

## 3. Results

### 3.1. Phytochemical and Antioxidant Analysis

The quantitative distribution of phytochemical compounds and their corresponding antioxidant capacities varied substantially across the evaluated plant extracts (Table 3). Spectrophotometric quantification revealed that *S. macrophylla* bark extract possessed the richest profile of total phenolics (97.67 mg GAE/g DM) and total flavonoids (12.42 mg QE/g DM). Conversely, the highest concentration of total tannins was detected in *A. mangium* bark extract, reaching a peak of 600.45 mg TAE/g DM, which was significantly higher than the levels observed in the other treatments. For comparison, *S. macrophylla* bark extract contained 355.82 mg TAE/g DM of tannins.

These distinct phytochemical yields directly influenced the free radical-scavenging performance of the extracts. The tannin-rich *A. mangium* extract demonstrated a superior capacity to quench DPPH radicals, closely followed by the *S. macrophylla* extract, which exhibited a robust antioxidant activity of 634.58 ± 15.84 µmol TE/g DM. On the other hand, black cumin essential oil yielded the lowest numerical values across all monitored phytochemical groups and consequently displayed relatively weak DPPH radical scavenging potential, despite harboring detectable trace amounts of phenolics, flavonoids, and tannins.

**Figure 1:**
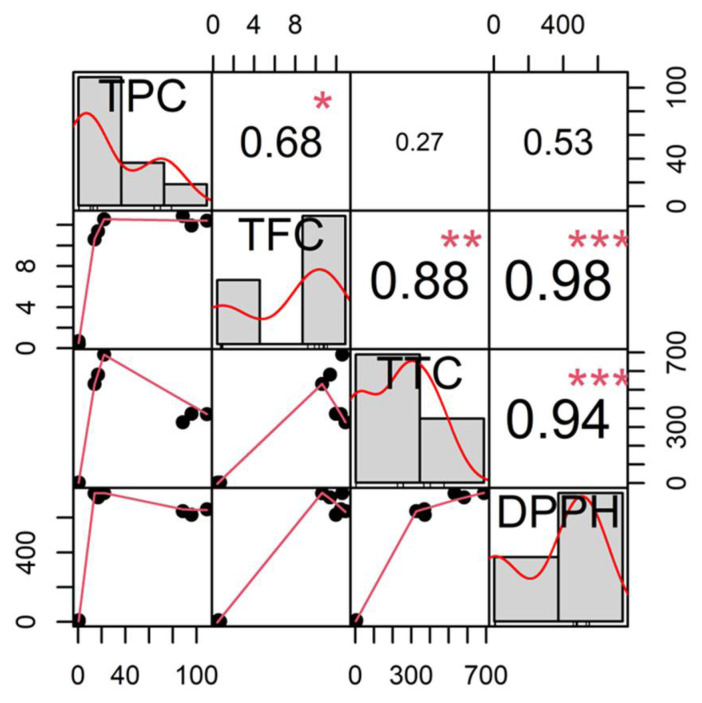
Pearson correlation matrix of phytochemical constituents and antioxidant activity. TPC = Total Phenolic Content, TFC = Total Flavonoid Content, TTC = Total Tannin Content, and DPPH = 2,2-Diphenyl-1-picrylhydrazyl radical scavenging activity. The upper panel shows Pearson correlation coefficients while the lower panel reports scatter plots. *, **, and *** indicate significance at *p* < 0.05, <0.01, and <0.001.

Pearson correlation analysis indicated predominantly positive interactions between the quantified phytochemical parameters and their antioxidant capacity (Figure 1). A robust linear relationship emerged between TPC and TFC (r = 0.88, *p* < 0.01), reflecting the major contribution of the flavonoid fraction to the total phenolic profile. Interestingly, TFC exhibited a near-perfect correlation with TTC (r = 0.98, *p* < 0.001), which aligns with the biochemical pathway where flavonoids act as primary precursors or structural building blocks for condensed tannin synthesis. Furthermore, the radical-scavenging activity against DPPH was strongly driven by the tannin fraction, as evidenced by a high correlation coefficient between TTC and DPPH values (r = 0.94, *p* < 0.001). This specific interaction underscores the superior efficiency of tannins in neutralizing free radicals within the matrix. Conversely, the association between TPC and DPPH was only moderate (r = 0.68, *p* < 0.05), whereas TPC and TTC showed a weak and non-significant correlation (r = 0.53, *p* > 0.05), suggesting that non-tannin phenolics did not contribute significantly to the observed antioxidant behavior.

### 3.2. Organoleptic and Physical Characteristics

The physical and organoleptic characteristics of the silage, including color, aroma, texture, and mold development, are presented in Table 4. LAB inoculation significantly affected the aroma and texture scores (*p* < 0.05), while the addition of plant extracts significantly influenced the color, aroma, texture, and mold contamination (*p* < 0.05). Among the evaluated plant extracts, acacia extract produced better organoleptic characteristics than the other treatments. The type of forage also significantly affected all physical and organoleptic parameters (*p* < 0.05), with *I. zollingeriana* silage showing superior preservation characteristics compared to other forages. Significant interactions between plant extracts and types of forage were observed in the color, aroma, and texture of the silage, whereas a significant three-way interaction between LAB, plant extracts, and types of forage was only found in the aroma parameter (*p* < 0.05).

The color scores ranged from 1.28 to 2.75, indicating color variation in the silage from yellowish-green to brownish-green. The aroma score for *I. zollingeriana* silage (1.25) reflected a better fresh sour aroma compared to other treatments. The texture scores ranged from 1.45 to 1.80, indicating that the silage generally maintained its physical integrity well. The fungal contamination scores ranged from 1.03 to 1.35. Silage supplemented with plant extracts consistently shows lower fungal contamination scores (1.03–1.08) compared to the untreated control (1.35). A significant LAB × phytobiotic × forage type interaction was detected only for aroma (*p* = 0.010), indicating that the response of aroma quality varied according to the specific combination of inoculation, phytobiotic source, and forage species. No significant three-way interactions were observed for color, texture, or mold (*p* > 0.05).

### 3.3. Fermentation Products

The product of silage fermentation is presented in Table 5. LAB inoculation significantly lowered the silage pH and increased lactic acid concentration (*p* < 0.05). Conversely, silage supplemented with plant extracts showed slightly higher pH values than the treatment without extract addition. The type of forage significantly affects lactic acid production, where leguminous silage produced higher lactic acid concentrations than Napier grass silage (*p* < 0.05). LAB inoculation also significantly affected NH_3_-N concentration (*p* < 0.05), resulting in higher NH_3_-N levels than the control without inoculation. Among the types of forage used, Napier grass silage produced a higher concentration of NH_3_-N compared to legume silage (*p* < 0.05). A significant LAB × phytobiotic × forage type interaction was detected for pH, NH_3_-N, and lactic acid concentration (*p* ≤ 0.05), demonstrating that the response of silage fermentation characteristics varied according to the specific combination of inoculant, phytobiotic source, and forage type. Therefore, the effects of the individual factors should be interpreted in the context of their combined interactions.

### 3.4. Nutritional Profiles

The interactive and independent effects of the evaluated phytobiotics and probiotics on the nutritional matrix of the final silages are detailed in Table 6. Inoculation with *L. plantarum* significantly preserved the dry matter (DM), crude protein (CP), and ether extract (EE) contents relative to the uninoculated control (*p* < 0.05). Phytobiotic supplementation also exerted a positive regulatory effect on the crude protein profile (*p* < 0.05), with the peak CP accumulation observed in experimental units treated with black cumin essential oil. Among the raw plant materials, *I. zollingeriana* silage consistently maintained the highest baseline CP levels. Regarding lipid fractions, the incorporation of *S. macrophylla* extract significantly enhanced the overall ether extract values (*p* < 0.05), with legume-based silages systematically yielding richer fat profiles than their Napier grass counterparts. The structural carbohydrate fractions were also strongly modulated by the silage additives. Probiotic *L. plantarum* inoculation led to a significant reduction in both crude fiber (CF) and acid detergent fiber (ADF) concentrations, while simultaneously shifting the neutral detergent fiber (NDF) values upward (*p* < 0.05). A mirrored pattern was induced by the phytobiotic supplementation, which significantly lowered the CF and ADF fractions (*p* < 0.05). Notably, the application of *A. mangium* extract triggered a significant elevation in the total NDF (*p* < 0.05). When comparing the botanical species, the legume-based silages were generally characterized by lower CF, ADF, and NDF compositions compared to the more lignified Napier grass silage.

The addition of *L. plantarum* did not exert any statistically significant influence on the total ash content (*p* > 0.05). In contrast, the phytobiotic interventions significantly drove down the overall ash percentages (*p* < 0.05), with the most pronounced reduction observed in the experimental units treated with black cumin essential oil. The specific macro-mineral profiles were also heavily modulated by the experimental additives. Probiotic inoculation with *L. plantarum* led to a significant reduction in the Ca concentrations (*p* < 0.05), while leaving the P levels statistically unaffected. Conversely, botanical extract supplementation acted as a positive regulator for mineral retention, significantly elevating both Ca and P concentrations (*p* < 0.05). Specifically, the inclusion of mahogany bark extract yielded the peak Ca accumulation, whereas the black cumin essential oil treatment was characterized by the highest P content. At the biomass level, legume-based silages systematically outperformed Napier grass silages in terms of inherent Ca and P status (*p* < 0.05). Highly significant three-way interactive effects (LAB × phytobiotic source × forage type) were successfully established across all monitored nutritional parameters (*p* < 0.05).

### 3.5. Amino Acid Profiles

The independent and interactive effects of the evaluated phytobiotics and probiotics on the essential amino acid (EAA) composition of the silages are summarized in Table 7. Inoculation with *L. plantarum* significantly elevated the concentrations of histidine, isoleucine, threonine, tryptophan, and valine (*p* < 0.05), while simultaneously driving down lysine and phenylalanine levels. Conversely, the probiotic treatment exerted no statistically significant impact on the leucine and methionine fractions. Regarding the botanical additives, phytobiotic supplementation markedly enhanced the overall essential amino acid pool (*p* < 0.05), with the sole exception of tryptophan, which underwent a significant reduction following extract incorporation. The inherent botanical characteristics of the raw materials also strongly influenced the amino acid yields. Napier grass silage was systematically characterized by the lowest baseline concentrations of histidine, lysine, threonine, tryptophan, and valine compared to the legume-based treatments (*p* < 0.05). Among the legumes, distinct variations emerged; *Indigofera zollingeriana* and *Gliricidia sepium* silages yielded the highest concentrations of lysine and methionine (*p* < 0.05). On the other hand, *Stylosanthes guianensis* silage displayed the most depleted profiles for isoleucine, leucine, methionine, and phenylalanine (*p* < 0.05). A significant three-way interaction (LAB × phytobiotic source × forage type) was established, confirming that the synchronization of these three experimental factors heavily modulated the final essential amino acid profiles (*p* < 0.05).

The distribution of non-essential amino acids in the ensiled biomass under the influence of phytobiotics and probiotics is compiled in Table 8. Inoculation with *L. plantarum* significantly diminished alanine and arginine fractions, while simultaneously triggering a significant elevation in cysteine, glycine, glutamic acid, and serine concentrations (*p* < 0.05). Beyond the probiotic effects, phytobiotic supplementation also played a pivotal role in modulating the non-essential amino acid profiles. Specifically, the incorporation of *Acacia mangium* extract markedly enriched the concentrations of alanine, glycine, glutamic acid, and tyrosine (*p* < 0.05). Conversely, *Swietenia macrophylla* (mahogany) bark extract enhanced aspartic acid accumulation but led to a significant contraction in alanine content (*p* < 0.05). In stark contrast to the bark extracts, black cumin essential oil supplementation significantly drove up cysteine, proline, and serine levels, while concurrently depleting the glycine and glutamic acid concentrations (*p* < 0.05). The botanical origin of the forage also exerted a strong independent effect on the final non-essential amino acid yields (*p* < 0.05). Interestingly, Napier grass silage was characterized by the highest baseline concentrations of proline and tyrosine, outperforming the legume-based silages (*p* < 0.05), A significant three-way interaction (LAB × phytobiotic source × forage type) was successfully established for nearly all monitored non-essential amino acid variables (*p* < 0.05), with the sole exception for aspartic acid, which remained statistically unaffected by the three-way interaction (*p* > 0.05).

### 3.6. Hierarchical Cluster Analysis of Amino Acid Profiles

**Figure 2:**
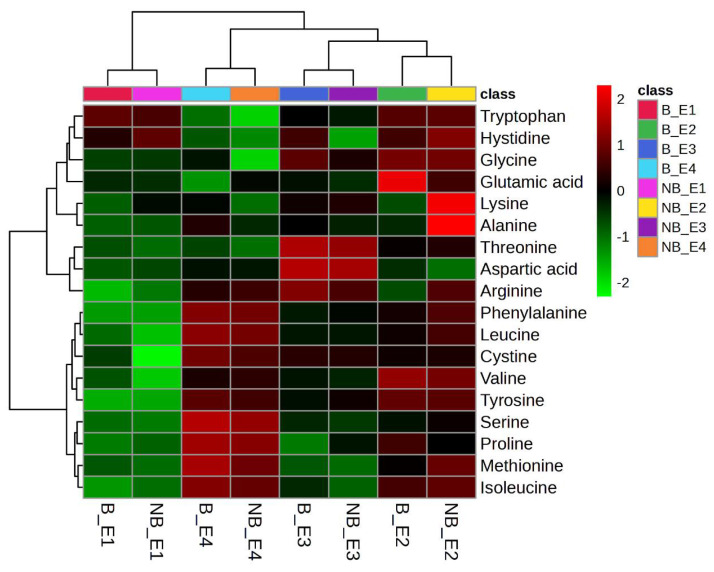
Cluster of treatment interactions with amino acid content. B: addition of lactic acid bacteria (LAB) inoculum; NB: without addition of lactic acid bacteria (LAB) inoculum; E1: Without extract; E2: Administration of acacia bark extract; E3: Administration of mahogany bark extract; E4: Administration of cumin essential oil.

Hierarchical cluster analysis was performed to systematically visualize the global variations in amino acid composition among the diverse silage treatments (Figure 2). The generated two-dimensional dendrogram and heatmap revealed distinct grouping patterns, establishing the phenotypic similarities and chemotaxonomic distances across the experimental units. Within the heatmap matrix, a divergent color scale was employed, where green-shaded tiles denote lower relative concentrations and red-shaded tiles signify higher abundance levels.

The horizontal dendrogram successfully segregated the treatments into two primary clusters, reflecting sharp metabolic differences between the fermented cohort (Group B) and the non-fermented reference group (Group NB). Notably, specific amino acid hotspots emerged within the topiary framework; glutamic acid, lysine, and alanine exhibited prominent high-intensity zones in LAB-inoculated silages that had been combined with *Acacia mangium* and *Swietenia macrophylla* bark extracts. In contrast, peripheral sub-clusters highlighted a coordinated depletion of tryptophan, histidine, and glycine across specific treatment lines, underscoring the selective amino acid remodeling driven by the interaction of probiotic inoculation and phytobiotic supplementation.

## 4. Discussion

Phytochemical screening of the *S. macrophylla* extract confirmed a rich profile of bioactive secondary metabolites, including flavonoids, tannins, triterpenoids, saponins, and alkaloids [30]. The robust antioxidant activity exhibited by this extract is fundamentally governed by its high concentration of phenolic compounds. Structurally, these molecules possess hydroxyl groups attached directly to aromatic rings, which function as efficient hydrogen atom donors capable of neutralizing unstable free radicals [31].

In the context of *S. macrophylla*, this potent radical-scavenging capacity is heavily driven by the presence of specific monomeric and oligomeric polyphenols, such as swietenemacrophyllanin, epicatechin, and catechin. The strong correlation between these phytochemical components and antioxidant performance was explicitly validated through the DPPH assay. Mechanistically, this colorimetric method evaluates the chemical reactivity of antioxidant compounds against stable 2,2-diphenyl-1-picrylhydrazyl radicals, which exhibit a characteristically intense violet coloration and a strong absorption peak at 517 nm. Upon accepting a hydrogen atom or an electron from the bark extract’s active constituents, the DPPH radical undergoes a stoichiometric reduction, causing a distinct color shift from deep purple to yellow that is directly proportional to the density of paired electrons [22].

*A. mangium* extract is characterized by a high abundance of polyphenolic secondary metabolites, predominantly in the form of condensed tannins (proanthocyanidins). When applied at judicious dietary dosages, these specialized polymers serve as effective functional feed additives in ruminant nutrition [2]. At the molecular level, the proanthocyanidins in *A. mangium* bark consist of recurring flavonoid subunits—primarily flavan-3-ols—interlinked with three principal dimeric structures: robinetinidol-(4α-8)-catechin, fisetinidol-(4β-8)-catechin, and the novel natural compound robinetinidol-(4β-8)-catechin [32]. This specific dimeric configuration, particularly the catechin moieties, plays a pivotal role in membrane-disrupting antimicrobial activities. Mechanistically, these polyphenols induce the cross-linking and subsequent precipitation of bacterial membrane proteins, thereby compromising cellular integrity [33]. Furthermore, the broad biological efficacy of these tannins is tightly linked to their cross-linking affinity with dietary proteins, forming stable tannin–protein complexes that effectively shield valuable nutrients from excessive ruminal microbial degradation. Beyond their antimicrobial and protein-sparing protection, the dense proanthocyanidin network within the *A. mangium* matrix governs its superior radical-scavenging performance compared to alternative botanical extracts. The underlying free radical-quenching efficiency of these extracts was previously highlighted by an exceptionally low half-maximal inhibitory concentration (IC_50_) value of 7.80 µg/mL [34]. Variations in antioxidant content may be influenced by fractionation methods, *Acacia* species differences, and environmental conditions where the plants grow [35].

In the present study, the essential oil derived from black cumin (*Nigella sativa*) yielded lower numerical values for total phenolics, flavonoids, and DPPH radical-scavenging capacity relative to the bark extracts. This apparent discrepancy does not necessarily imply a lack of functional potency, but rather reflects the chemical limitations of conventional aqueous-methanol colorimetric assays in fully capturing the bioactivity of highly volatile, hydrophobic lipophilic fractions. Despite these lower baseline in vitro antioxidant readings, the practical efficacy of *N. sativa* essential oil as a potent antimicrobial stabilizing agent for silage preservation aligns with the findings of Susanto et al. [16]. The antibacterial properties inherent to black cumin serve as a defensive barrier against spoilage microorganisms, effectively suppressing undesirable bacterial proliferation by disrupting lipid bilayer membranes, thereby preserving the physical integrity and nutritional matrix of the ensiled forage [36]. The protective performance of *N. sativa* is deeply rooted in its specialized phytochemical architecture. The essential oil serves as a matrix for critical bioactive compounds, notably alpha-hederin, nigellidine, nigellicine, nigellimine, thymoquinone, and thymohydroquinone [37]. While these metabolites exhibit a broad spectrum of pharmacological effects—ranging from anti-inflammatory to tissue-protective activities—thymoquinone stands out as the primary lipophilic driver responsible for both localized antioxidant defense and cellular stabilization [38]. Additionally, longifolene exhibits antioxidant and antibacterial activity, while thymol has strong antimicrobial properties [39].

The pronounced statistical interdependencies observed among the phenolic, flavonoid, and tannin pools underscore a shared and highly synchronized metabolic lineage. Biologically, the near-perfect alignment between flavonoid and tannin concentrations validates the established metabolic paradigm wherein flavan-3-ols and other monomeric flavonoids operate as the fundamental structural building blocks for condensed tannin biosynthesis.

When evaluating the functional outputs of these secondary metabolites, the tannin fraction emerged as the principal architect of the extracts’ radical-scavenging capacity. The strong interaction between TTC and DPPH values corroborates earlier observations [40,41], which demonstrated that the high degree of polymerization and multiple hydroxyl-rich domains in condensed tannins exponentially magnify their free-radical quenching efficiency. By contrast, the broader and more generalized phenolic pool (TPC) exhibited only a moderate influence on DPPH reduction [42], coupled with a notably weak statistical association with total tannins. This disconnect suggests a distinct metabolic divergence within the plant matrix; a substantial proportion of the quantified total phenolics likely consists of simple phenolic acids or non-polymeric flavonoids that do not actively participate in tannin construction [43]. Consequently, while the general phenolic content provides baseline cellular protection, it is the specific structural complexity of the polymerized flavonoid–tannin network that dictates the ultimate antioxidant superiority of the extracts.

Color is an important indicator of the quality of silage because it reflects the success of the preservation process. Biomass that is well-preserved generally retains the green pigment of fresh plants after the ensiling process. The color change in silage is related to aerobic respiration in the early phase of ensiling, which oxidizes soluble carbohydrates into CO_2_, H_2_O, and heat, thereby accelerating the browning of the forage [24]. In addition to the increase in temperature during fermentation, the color characteristics of silage are also influenced by the chemical composition of the raw materials used [44]. The color variations observed between treatments are likely related to the degree of chlorophyll degradation during the ensilage process, as the loss of green pigment is a major factor determining color changes in mature silage [45].

The addition of *L. plantarum* increases lactic acid production during the ensiling process, contributing to the formation of a sour aroma characteristic of high-quality silage. These findings are in line with those of Wang et al. [46], who stated that the conversion of structural carbohydrates and soluble sugars into organic acids by microbes is the main factor determining the sensory characteristics of silage. In this study, *Indigofera* silage produced a fresh sour aroma with the best score compared to other silages, indicating that fermentation proceeded well and was associated with a high concentration of lactic acid produced. The variation in aroma among treatments is likely influenced by the interaction between LAB inoculation, plant extracts, and the type of forage. Additionally, the availability of fermentable substrates and the buffering capacity of the raw materials are known to play a crucial role in determining the fermentation pathway [47]. The differences in the characteristics of these raw materials can explain the variation in aroma observed between leguminous silage and elephant grass. For example, tropical grass silage tends to produce higher concentrations of acetic acid due to differences in fermentation pathways, which in turn gives a sharper and more pungent aroma compared to silage dominated by lactic acid [48].

Structurally, the physical integrity and tactile firmness of the resulting silage are heavily dictated by the initial moisture content of the harvested biomass. According to Zulkarnaim et al. [49], high moisture during the ensiling phase generally accelerates the breakdown of plant tissues, leading to a noticeable loss of cellular turgidity and resulting in a softer feed texture. The presence of slime, particularly observed in the Napier grass silage, serves as a direct biomarker for microbial spoilage. The untreated control silages suffered the highest degree of slime accumulation and structural deterioration. This degradation occurred because the silo environment lacked the rapid acidification normally provided by lactic acid bacteria, as well as the targeted antimicrobial suppression derived from the bioactive plant extracts. Conversely, the simultaneous integration of LAB and plant extracts successfully mitigated these spoilage pathways. By rapidly dropping the pH to arrest undesirable microbial growth and directly neutralizing the opportunistic flora responsible for slime formation, the additives effectively preserved the physical architecture of the silage. These protective dynamics closely corroborate the preservation mechanisms outlined by Azzahra et al. [50], reinforcing the necessity of plant extracts and LAB supplementation in maintaining the structural quality of ensiled tropical forages.

The complete absence of fungal growth in the extract-treated silages highlights the strong antifungal properties of these plant additives. Generally, fungal development in silage is triggered by the presence of trapped oxygen and residual moisture inside the silo [45]. While proper physical compaction is the primary method to minimize air exposure, the addition of plant extracts provides a vital secondary barrier against aerobic spoilage. At the cellular level, these plant-derived compounds directly inhibit fungal survival. Their main mechanism involves disrupting the fungal cell membranes and preventing the formation of new cell walls [51]. By controlling these spoilage microorganisms, the extracts help prevent the breakdown of lactic acid and the subsequent rise in pH. Consequently, this targeted antifungal action plays a key role in maintaining a stable, oxygen-free environment, thereby ensuring the overall fermentation quality of the silage [5].

The recorded pH values in this study ranged from 4.77 to 5.36, which exceeds the standard optimal threshold of strictly below 4.2 for well-preserved silage [52]. However, this elevated pH is a well-documented characteristic of legume-based silages, which inherently struggle to drop their pH below 4.5 [53]. This resistance to acidification is primarily caused by their high crude protein content, which creates a strong natural buffering capacity inside the silo [54]. Because prolonged high pH levels can allow spoilage bacteria to survive and multiply [55], lowering the pH as much as possible remains a priority. Despite this natural buffering challenge, the specific combination of the LAB inoculum and *Acacia* extract successfully drove the pH down to 4.64 in the *Indigofera* silage. This effective pH reduction is a direct result of bacterial metabolism. As the inoculated *L. plantarum* actively ferments the available plant sugars, it produces a steady accumulation of lactic acid, which gradually acidifies the silo environment and helps preserve the forage [56,57].

Effective LAB inoculation can enhance the release and breakdown of sugars in the early stages of ensilage, resulting in increased lactic acid production [56]. In this study, LAB-inoculated Indigofera silage showed the highest lactic acid concentration; however, its value decreased after the addition of acacia extract. These findings indicate that plant extracts rich in tannins can affect the course of fermentation. The antibacterial properties of tannins can disrupt the integrity of bacterial cell walls [58], thereby causing plant extracts to inhibit lactic acid bacteria and limit their capacity to produce acid. In addition to feed additives, the intrinsic characteristics of forages also play an important role in determining fermentation outcomes. Optimal lactic acid production generally requires an initial WSC content of 3–5% [52]. However, the success of fermentation is not only determined by the amount of available sugar but also by the accessibility of the substrate to microorganisms. The high fiber content in forages can limit the utilization of carbohydrates stored in the plant cell wall matrix, thereby reducing fermentation efficiency and lactic acid production [59].

In silage evaluation, the accumulation of NH_3_-N is one of the main indicators that shows the ongoing degradation of protein by undesirable microorganisms [2]. Unexpectedly, the inoculation of BAL in this study caused a significant increase in ammonia levels. These results differ from the typical fermentation pattern, where L. plantarum usually accelerates the decrease in pH, allowing protein-degrading bacteria to be inactivated quickly and preventing ammonia accumulation [60]. However, similar results to this study were reported by Yang et al. [61], who found that *L. plantarum* produced higher NH_3_-N compared to the control, even though lactic acid fermentation was more intensive. The activity of proteolytic enzymes in plants can still occur within the pH range of 5-6, allowing protein degradation to continue during the ensiling process [29,62]. Furthermore, in this study, the final pH of the silage was relatively high. These conditions are likely not sufficient to effectively inhibit the activity of plant proteases or the growth of undesirable proteolytic microorganisms. Consequently, the deamination process of amino acids can continue, leading to a higher accumulation of NH_3_-N.

Fortunately, the inclusion of *Acacia* extract proved effective in mitigating severe protein loss. The lower NH_3_-N levels observed in this specific treatment confirm that its rich tannin content successfully bound to the plant proteins, shielding them from microbial degradation. However, the protective power of tannins is highly dependent on the acidity of the silo. When the silage pH remains too high, the chemical bonds between tannins and proteins weaken because the phenol groups within the tannins become ionized [63]. This pH dependency perfectly explains the extensive protein breakdown observed in the Napier grass silage. Because the Napier grass experienced a very slow and insufficient drop in pH, the opportunistic spoilage bacteria were able to survive longer. Simultaneously, this elevated pH prevented the tannins from binding effectively to the proteins. This unfortunate combination of active spoilage microbes and unprotected plant proteins could result in excessive ammonia production and overall poor fermentation quality.

The effectiveness of tannins in protecting proteins is strongly influenced by environmental pH. Tannin–protein complexes are generally most stable under mildly acidic conditions (approximately pH 3.5–5.0), where hydrophobic interactions and hydrogen bonding are maximized [64]. As pH increases toward neutrality, ionization of functional groups alters these interactions, reducing binding affinity and facilitating partial dissociation of the complexes [18]. In the present study, silage pH ranged from 4.77 to 5.36, indicating that tannin–protein binding was likely retained to some extent but not at its maximum stability. Consequently, although *Acacia mangium* extract reduced protein degradation and improved amino acid preservation, the persistence of moderate pH conditions may have permitted residual proteolytic activity and NH_3_-N accumulation. This pH-dependent behavior provides a plausible explanation for the partial effectiveness of the tannin-rich extract observed in this study.

Although LAB inoculation is generally associated with reduced proteolysis and lower NH_3_-N accumulation during ensiling, contradictory findings have occasionally been reported. The inhibitory effect of LAB on proteolysis largely depends on achieving a sufficiently rapid decline in pH to suppress plant proteases and deaminating microorganisms. In the present study, the relatively high final pH observed in certain forage silages may have allowed residual proteolytic activity to persist [65,66]. This phenomenon may be particularly relevant in tropical legumes, which possess high buffering capacities attributable to their elevated protein and mineral contents, thereby slowing acidification. This is supported by the study of Castro-Montoya and Dickhoefer [67], which reported that several forage species, particularly legumes, naturally contain higher levels of basic compounds such as proteins (acting as nitrogen sinks) and minerals, making them more resistant to pH decline and requiring significantly greater amounts of acid to reach a stable pH. In addition, strain-dependent differences in *L. plantarum* may influence the extent of proteolysis inhibition [68]. Therefore, the increased NH_3_-N concentration observed in this study likely resulted from the combined effects of forage buffering capacity and incomplete suppression of proteolytic processes from the combined effects of phytobiotics and probiotics.

An apparent discrepancy was observed between the preservation of certain amino acids and the increased NH_3_-N concentration following LAB inoculation. Although NH_3_-N is widely regarded as an indicator of proteolysis and amino acid deamination, amino acid preservation and NH_3_-N formation do not necessarily exhibit a direct inverse relationship. The persistence of a relatively high silage pH in different tropical legumes may have permitted partial deamination to continue while simultaneously allowing the preservation of specific amino acids through improved fermentation conditions. Moreover, the naturally occurring bioactive compounds present in tropical legumes, together with the phytobiotic extracts supplemented in the present study, may have contributed to the selective protection of amino acids during the ensiling process. Our recent studies demonstrated that several of these plant extracts possess a high affinity for binding free amino acids and can inhibit amino acid deamination by suppressing the activity of glutamate dehydrogenase, thereby reducing the conversion of amino acids into ammonia [17,18]. Consequently, the combined application of LAB inoculants and phytobiotics appeared to enhance the preservation of selected amino acids rather than completely suppressing nitrogen degradation pathways. These findings suggest that improved amino acid retention can occur concurrently with partial nitrogen degradation, particularly in tropical forage legumes characterized by high buffering capacity and incomplete acidification during the ensiling process.

The overall improvement in the nutritional profile of the inoculated silages clearly highlights the value of *L. plantarum* in maximizing fermentation efficiency. By driving a rapid drop in pH, the lactic acid bacteria effectively shut down protein-degrading enzymes, leading to much better crude protein retention in the final feed [69]. Interestingly, while the bacteria protect these valuable proteins, their active fermentation has the opposite and highly beneficial effect on structural carbohydrates. The steady production of organic acids accelerates the breakdown of tough crude fiber, making the silage more digestible [62]. However, this intense acidification process also directly explains the observed decrease in Ca levels in the LAB-treated silages. As the fermentation environment becomes highly acidic, structural minerals like calcium tend to become highly soluble. It is most likely that the calcium was converted into soluble calcium lactate and subsequently lost during the ensiling period.

In addition to the microbial inoculants, the specific type of plant extract used significantly shaped the final nutrient profile of silage. Plant extracts rich in bioactive compounds, such as flavonoids and tannins, function as natural chemical preservatives. They actively suppress opportunistic spoilage microbes, which prevents the unwanted breakdown of valuable plant proteins and helps retain essential nitrogen within the feed. This protective mechanism is further reinforced by the strong antimicrobial properties of black cumin essential oil, which protects the overall fermentation environment from harmful bacterial overgrowth [70]. By successfully limiting protein degradation and favorably influencing plant cell wall breakdown, these plant extracts ensure a much higher quality of the finished feed [62].

The nutritional value of silage is fundamentally shaped by the characteristics of the forage. This variation stems primarily from the natural differences in plant cell wall structures and overall fermentability. For example, the specific lignin and cellulose content within a plant species directly dictates its fiber digestibility [71]. Furthermore, there is a clear nutritional divide between plant families; legume-based forages such as *Indigofera* and *Gliricidia* naturally provide significantly higher crude protein levels than tropical grasses such as Napier grass. The success of fermentation depends heavily on the initial plant composition when microbial inoculants are introduced. The interaction between the specific forage type and LAB determines the overall efficiency of microbial activity. As bacteria actively consume soluble nutrients during fermentation, the remaining unfermentable structural components of the plant, specifically the ash and lignin fractions, naturally become more concentrated in the final silage product.

Combining LAB inoculants with plant extracts created a strong synergistic effect that significantly improved the overall silage quality. Specifically, pairing mahogany extract with LAB reduced the crude fiber content more effectively than using bacterial treatment alone. By suppressing undesirable microbes, bioactive plant compounds likely reduced microbial competition inside the silo. This allows beneficial lactic acid bacteria to dominate the environment and break down structural fibers with much greater efficiency [72]. A similarly complementary relationship was observed in protein preservation. When black cumin essential oil was applied to *Gliricidia*, the resulting silage retained the highest crude protein concentration across all experimental treatments. This exceptional nutrient retention is directly driven by the specialized secondary metabolites found in cumin oil, which actively shut down the bacterial enzymes responsible for protein degradation during the fermentation period [73].

Strong interactions observed among the LAB inoculants, plant extracts, and forage highlight the practical value of combining these additives. When applied together, probiotics and phytobiotics work synergistically to safeguard essential nutrients, such as proteins and minerals, while simultaneously breaking down tough structural fibers. This targeted approach is especially critical for tropical farming systems. Because tropical regions possess a wide diversity of forage resources, producers have the unique opportunity to match specific botanical extracts with local grasses or legumes to maximize fermentation success [52]. Moving forward, acknowledging that there is no single universal additive is key; carefully customizing the combination of inoculants and plant extracts to suit the specific crop will be essential for producing high-quality silage and improving overall livestock performance.

Preserving the precise amino acid profile of ensiled forage is a delicate process that depends heavily on the interaction between the lactic acid bacteria, the added plant extracts, and the raw material itself. In this protective relationship, the LAB inoculant serves as the foundation by rapidly stabilizing the fermentation environment [74].

When used with LAB, plant extracts may provide an additional layer of protection against protein degradation during ensiling. The bioactive compounds within these extracts, such as flavonoids and tannins, have been reported to suppress proteolytic microorganisms and inhibit protease activity, thereby reducing amino acid degradation and NH_3_-N formation [17,75]. As the abundance and composition of these protective metabolites differ significantly among plant species, the intrinsic biochemical characteristics of the harvested biomass also play an important role in determining protein preservation during fermentation. The combined application of LAB and plant extracts may represent a promising strategy for improving amino acid retention by integrating rapid acidification with the antimicrobial and antiproteolytic properties of plant secondary metabolites.

The practical benefits of LAB and plant extracts are most evident in the final amino acid yield. Silages treated exclusively with the LAB inoculant retained noticeably fewer essential and non-essential amino acids compared to those receiving the combined treatment. This measurable difference confirms that bacterial acidification alone is not always sufficient to fully secure nutrients in plants. By introducing additional plant extracts, specifically polyphenols and saponins, into the silo, a vital secondary defense is provided. These compounds work alongside the active fermentation process to effectively halt protein breakdown, resulting in a much richer and well-preserved amino acid profile [76].

Fundamentally, this successful amino acid retention is driven by the strong antimicrobial and antioxidant properties of plant extracts. The distinct phenolic compounds present in both the cumin essential oil and *Acacia* bark extract actively suppress the opportunistic bacteria responsible for deamination [77]. Therefore, pairing these natural plant extracts with standard LAB inoculants provides a highly practical and effective strategy for safeguarding protein quality. By simultaneously preventing protein breakdown and optimizing the fermentation environment, this combined approach can significantly elevate the nutritional value of feed in livestock production systems. To build on these promising results, future studies should evaluate how different local forages and plant extract combinations maintain this amino acid stability over extended, long-term storage periods.

The combination of BAL with acacia and mahogany extracts consistently improves the overall preservation of amino acids. This combination creates fermentation conditions associated with lower nitrogen loss and higher amino acid retention. Conversely, the treatment that did not have that combination experienced significant losses of certain amino acids, particularly tryptophan, histidine, and glycine. This study shows that these amino acids are likely very susceptible to degradation when fermentation conditions are less supportive. The observed pattern confirms that the selection of the right combination of bacterial inoculants and plant extracts is an important factor in controlling protein turnover and maintaining the amino acid profile in silage [78]. Although this study shows that the combination of LAB and plant extracts can reduce the accumulation of NH_3_-N and preserve amino acids, the underlying microbial mechanisms of this process were not directly evaluated. Further research needs to integrate microbial enumeration analysis and characterization to verify the biological mechanisms involved in the inhibition of deamination.

Evidence supports this preservation strategy for maximizing silage quality. Specifically, introducing targeted LAB strains like *L. plantarum* is known to effectively shield critical amino acids such as lysine and methionine from severe proteolytic breakdown [79]. This bacterial action is perfectly complemented by the phenolic compounds naturally present in *Acacia* and mahogany bark extracts. By acting as strong natural antimicrobials and antioxidants, these plant extracts directly reinforce amino acid stability throughout the entire fermentation period [62]. The distinct clustering patterns observed in the uninoculated control groups point to a much more erratic fermentation process. Without the dominant presence of LAB, unpredictable indigenous microbial populations take over the silo environment, triggering alternative metabolic pathways that rapidly degrade the available amino acids [80]. Actively controlling the silo environment through the strategic combination of bacterial inoculants and plant-derived bioactives offers a practical method for significantly upgrading livestock nutrition. To continue refining this approach, subsequent investigations should focus on mapping the precise biochemical interactions between various LAB strains and different botanical extracts across long-term storage scenarios.

The true nutritional value of silage is heavily reflected in its final amino acid profile. When the fermented feed successfully retains high levels of essential amino acids, particularly lysine and methionine, provides clear evidence that the plant proteins were effectively shielded from breakdown, resulting in superior livestock feed [52]. If these nutrient levels drop, it is a direct sign that undesirable microbes have actively degraded the forage. Because of this dynamic, measuring amino acid retention serves as an excellent benchmark for evaluating overall fermentation efficiency. The positive outcomes associated with LAB inoculation perfectly illustrate this relationship. By rapidly dominating the silo environment, the bacterial inoculant fundamentally shifts the microbial community. This shift actively suppresses the specific destructive enzymes that would otherwise cause amino acid loss, thereby securing the stability of the feed [78].

Beyond biological inoculants, the overall ensiling environment heavily dictates how specific amino acids, such as lysine and arginine, are distributed and utilized for energy metabolism [81,82]. The preservation of lysine, histidine, valine, and tryptophan is nutritionally important because these amino acids contribute to microbial protein synthesis, growth performance, milk protein production, and overall nitrogen utilization efficiency in ruminants [83]. The profound impact of this process is most evident when comparing the fermented forage (Group B) directly with the unfermented forage (Group NB). The data confirms that proper fermentation acts as a biological processor, naturally unlocking and increasing the availability of essential plant-based proteins [84,85].

Interestingly, the control treatments without LAB and *Acacia* bark extract exhibited a largely uniform amino acid profile. This homogeneity likely reflects a standard, unregulated breakdown process where no specific nutrients are actively shielded from degradation. In contrast, intentionally applying targeted microbial and botanical treatments alters this composition, directly improving the overall bioavailability of the protein for the animal [86]. Ultimately, fine-tuning these fermentation strategies by pairing the right microbial strains with protective plant extracts will be the definitive key to consistently producing high-quality, nutrient-dense livestock feed.

Despite the promising effects observed in this study, several limitations should be acknowledged. First, phytobiotic extracts were compared on an equal fresh-weight basis rather than equivalent phytochemical concentrations. Second, the mechanisms underlying the increased NH_3_-N concentrations were inferred from fermentation characteristics and were not directly assessed through protease activity measurements. Third, the experiment was conducted under controlled laboratory-scale ensiling conditions. Future studies should investigate proteolytic enzyme activity and standardized phytochemical dosing to better elucidate the mechanisms involved.

## 5. Conclusions

The integration of *Lactiplantibacillus plantarum* inoculum and phytobiotics improved the physical characteristics, fermentation profile, and nutrient preservation of silage while reducing pH. Among the treatments evaluated, the combination of L. plantarum and Acacia bark extracts applied to *Indigofera zollingeriana* silage produced the most favorable overall response, as evidenced by improved physical quality, reduced visible fungal contamination, lower NH_3_-N accumulation, and greater retention of essential amino acids, including histidine, lysine, tryptophan, and valine. The significant three-way interaction among LAB inoculation, phytobiotic source, and forage type indicates that the effectiveness of phytobiotic supplementation depends on both the inoculant and forage species. These findings suggest that combining *L. plantarum* with tannin-rich Acacia bark extract represents an effective strategy for improving the fermentation quality and nutritional preservation of tropical silage. However, further studies integrating metagenomic and metabolomic approaches are required to elucidate the interactions between silage microorganisms and plant secondary metabolites and to verify the mechanisms underlying the reduction in deamination during ensiling.

## Figures and Tables

**Table 1:** Chemical composition of green fodder used as silage.

Forage Type	DM (%)	Protein (% DM)	Fiber (% DM)	NDF (% DM)	ADF (% DM)
*Pennisetum purpureum*	24.30 ± 0.14	14.22 ± 0.01	31.34 ± 0.01	84.45 ± 0.01	56.16 ± 0.01
*Indigofera zollingeriana*	29.60 ± 0.28	23.30 ± 0.03	25.47 ± 0.04	53.28 ± 0.08	39.19 ± 0.06
*Gliricidia sepium*	29.20 ± 0.28	21.72 ± 0.04	32.13 ± 0.06	57.92 ± 0.11	44.67 ± 0.09
*Stylosanthes guianensis*	41.50 ± 0.71	18.07 ± 0.02	42.97 ± 0.07	72.26 ± 0.12	59.08 ± 0.10

**Table 2:** Indicators of physical quality assessment of silage.

Score	Silage			
	Color	Aroma	Texture	Presence of Fungi
1	Yellowish-green	Fresh sour	Not lumpy and not slimy	None
2	Brownish-green	Sour	Slightly lumpy and slightly slimy	Few
3	Blackish-brown	Less sour	Lumpy and slimy	Many on the surface
4	Black	Rotten	Much lumpy and slimy	Many on all surfaces

**Table 3:** The content of phytochemicals and antioxidant activity of acacia bark extract, mahogany bark extract, and cumin essential oil.

Extract	TTC (mg TAE/g DM)	TFC (mg QE/g DM)	TPC (mg GAE/g DM)	DPPH (µmol TE/g DM)
Acacia bark	600.45 ± 80.75 ^c^	11.52 ± 0.98 ^b^	17.58 ± 4.08 ^b^	733.09 ± 13.86 ^c^
Mahogany bark	355.82 ± 25.85 ^b^	12.42 ± 0.47 ^c^	97.67 ± 5.94 ^c^	634.58 ± 15.84 ^b^
Cumin essential oil	2.78 ± 0.57 ^a^	0.53 ± 0.08 ^a^	0.52 ± 0.03 ^a^	5.35 ± 2.05 ^a^

Values are expressed as mean ± SD. Different superscript letters within the same column indicate significant differences among extracts (*p* < 0.05). TPC = Total Phenolic Content, TFC = Total Flavonoid Content, TTC = Total Tannin Content, and DPPH = 2,2-Diphenyl-1-picrylhydrazyl radical scavenging activity; TAE = tannic acid equivalent; QE = quercetin equivalent; GAE = gallic acid equivalent; TE = Trolox equivalent; DM = dry matter.

**Table 4:** The effect of using phytobiotics and probiotics on the physical quality of silage.

Treatment Factors	Color	Aroma	Texture	Mold
**LAB inoculation treatments**				
Non-LAB	1.75 ± 0.64	1.96 ± 0.82 ^b^	1.79 ± 0.34 ^b^	1.18 ± 0.21
LAB	1.80 ± 0.78	1.74 ± 0.83 ^a^	1.55 ± 0.33 ^a^	1.09 ± 0.12
**Extract source treatments**				
Non-Extract	1.50 ± 0.40 ^a^	2.13 ± 1.06 ^c^	1.78 ± 0.48 ^b^	1.35 ± 0.21 ^b^
Acacia extract	1.93 ± 0.93 ^b^	1.63 ± 0.79 ^a^	1.45 ± 0.25 ^a^	1.03 ± 0.07 ^a^
Mahogany extract	1.80 ± 0.76 ^b^	1.78 ± 0.78 ^ab^	1.70 ± 0.30 ^b^	1.08 ± 0.10 ^a^
Cumin essential oil	1.88 ± 0.69 ^b^	1.88 ± 0.69 ^b^	1.75 ± 0.29 ^b^	1.08 ± 0.10 ^a^
**Forage type treatments**				
*Pennisetum purpureum*	2.75 ± 0.74 ^c^	3.08 ± 0.56 ^c^	1.75 ± 0.51 ^c^	1.28 ± 0.23 ^b^
*Indigofera zollingeriana*	1.28 ± 0.10 ^a^	1.25 ± 0.20 ^a^	1.65 ± 0.15 ^ab^	1.10 ± 0.15 ^a^
*Gliricidia sepium*	1.40 ± 0.21 ^a^	1.58 ± 0.08 ^b^	1.80 ± 0.33 ^c^	1.05 ± 0.09 ^a^
*Stylosanthes guianensis*	1.68 ± 0.23 ^b^	1.50 ± 0.32 ^b^	1.48 ± 0.26 ^a^	1.10 ± 0.15 ^a^
**SEM**	0.065	0.074	0.046	0.027
***p*** **-value**				
LAB inoculation	0.531	0.007	0.004	0.087
Extract source	0.001	<0.001	0.021	<0.001
Forage type	<0.001	<0.001	0.029	0.012
LAB × Extract	0.344	0.202	0.356	0.652
LAB × Forage	0.001	0.292	0.412	0.765
Extract × Forage	<0.001	0.003	<0.001	0.941
LAB × Extract × Forage	0.472	0.010	0.869	0.964

Different superscript letters in the same column indicate significant effects (*p* < 0.05). SEM = standard error of the mean. LAB = lactic acid bacteria. The *p*-values represent the main effects of LAB inoculation, extract source, forage type, and their interactions.

**Table 5:** The effect of using phytobiotics and probiotics on silage product fermentability.

Treatment Factors	pH	NH_3_-N (mM)	Lactic Acid (g kg^−1^)
**LAB inoculation treatments**			
Non-LAB	5.04 ± 0.39 ^b^	4.59 ± 0.51 ^a^	23.88 ± 1.92 ^a^
LAB	4.93 ± 0.39 ^a^	5.80 ± 0.64 ^b^	29.97 ± 2.56 ^b^
**Extract source treatments**			
Non-Extract	4.84 ± 0.16 ^a^	5.11 ± 0.67 ^b^	33.57 ± 2.73 ^c^
Acacia extract	4.88 ± 0.08 ^b^	4.91 ± 0.53 ^a^	23.74 ± 1.39 ^a^
Mahogany extract	5.10 ± 0.50 ^c^	5.78 ± 0.62 ^c^	25.87 ± 2.54 ^b^
Cumin essential oil	5.13 ± 0.58 ^d^	4.97 ± 0.59 ^ab^	24.54 ± 2.33 ^a^
**Forage type treatments**			
*Pennisetum purpureum*	4.95 ± 0.11 ^c^	10.67 ± 1.55 ^d^	7.34 ± 0.61 ^a^
*Indigofera zollingeriana*	5.36 ± 0.62 ^d^	2.62 ± 0.33 ^a^	38.69 ± 1.10 ^c^
*Gliricidia sepium*	4.87 ± 0.14 ^b^	3.67 ± 0.39 ^b^	30.96 ± 1.94 ^b^
*Stylosanthes guianensis*	4.77 ± 0.09 ^a^	3.84 ± 0.19 ^c^	30.73 ± 1.83 ^b^
**SEM**	0.031	0.285	1.138
***p*** **-value**			
LAB inoculation	<0.001	<0.001	<0.001
Extract source	<0.001	<0.001	<0.001
Forage type	<0.001	<0.001	<0.001
LAB × Extract	<0.001	<0.001	<0.001
LAB × Forage	0.021	<0.001	<0.001
Extract × Forage	<0.001	<0.001	<0.001
LAB × Extract × Forage	0.002	<0.001	<0.001

Different superscript letters in the same column indicate significant effects (*p* < 0.05). SEM = standard error of the mean. LAB = lactic acid bacteria. The *p*-values represent the main effects of LAB inoculation, extract source, forage type, and their interactions.

**Table 6:** The effect of using phytobiotics and probiotics on the nutrient content of silage.

Treatment Factors	DM (%)	CP (% DM)	EE (% DM)	CF (% DM)	Ash (% DM)	NDF (%)	ADF (%)	Ca (%)	P (%)
**LAB inoculation treatments**									
Non-LAB	91.23 ± 0.25 ^a^	18.07 ± 0.79 ^a^	6.92 ± 0.26 ^a^	23.25 ± 0.75 ^b^	20.71 ± 1.04	37.43 ± 0.48 ^a^	41.22 ± 1.21 ^b^	1.21 ± 0.05 ^b^	0.65 ± 0.02
LAB	91.55 ± 0.26 ^b^	18.73 ± 0.72 ^b^	7.11 ± 0.31 ^b^	22.66 ± 0.82 ^a^	20.22 ± 1.13	38.25 ± 0.52 ^b^	40.12 ± 1.29 ^a^	1.17 ± 0.06 ^a^	0.66 ± 0.02
**Extract source treatments**									
Non-Extract	88.28 ± 0.22 ^a^	13.39 ± 0.96 ^a^	4.78 ± 0.34 ^a^	30.14 ± 1.01 ^d^	34.13 ± 0.51 ^d^	38.04 ± 0.62 ^b^	52.49 ± 1.69 ^c^	0.63 ± 0.04 ^a^	0.42 ± 0.02 ^a^
Acacia extract	90.65 ± 0.33 ^b^	16.54 ± 0.64 ^b^	7.96 ± 0.41 ^c^	23.21 ± 0.99 ^c^	15.07 ± 1.16 ^b^	41.94 ± 0.84 ^c^	37.43 ± 1.84 ^b^	1.14 ± 0.06 ^b^	0.61 ± 0.04 ^b^
Mahogany extract	92.63 ± 0.19 ^c^	16.15 ± 0.84 ^b^	9.59 ± 0.34 ^d^	20.51 ± 0.94 ^b^	27.07 ± 0.94 ^c^	37.82 ± 0.44 ^b^	34.84 ± 1.26 ^a^	1.65 ± 0.09 ^d^	0.66 ± 0.02 ^c^
Cumin essential oil	94.02 ± 0.23 ^d^	27.52 ± 0.94 ^c^	5.71 ± 0.23 ^b^	17.96 ± 0.95 ^a^	5.61 ± 0.49 a	33.56 ± 0.52 ^a^	37.92 ± 1.45 ^b^	1.33 ± 0.04 ^c^	0.92 ± 0.01 ^d^
**Forage type treatments**									
*Pennisetum purpureum*	90.80 ± 0.20 ^a^	10.40 ± 0.73 ^a^	4.22 ± 0.20 ^a^	34.70 ± 0.65 ^d^	19.14 ± 1.41 ^b^	40.95 ± 0.45 ^c^	51.62 ± 0.94 ^c^	0.58 ± 0.03 ^a^	0.46 ± 0.03 ^a^
*Indigofera zollingeriana*	93.64 ± 0.34 ^b^	24.76 ± 0.85 ^d^	9.59 ± 0.34 ^d^	14.75 ± 0.70 ^a^	23.86 ± 1.29 ^c^	38.39 ± 0.78 ^b^	27.53 ± 0.86 ^b^	1.26 ± 0.05 ^b^	0.72 ± 0.03 ^c^
*Gliricidia sepium*	90.59 ± 0.18 ^a^	21.95 ± 0.88 ^c^	9.15 ± 0.18 ^c^	17.08 ± 0.45 ^b^	15.33 ± 1.41 ^a^	35.98 ± 0.96 ^a^	28.85 ± 0.82 ^a^	1.41 ± 0.05 ^c^	0.79 ± 0.02 ^d^
*Stylosanthes guianensis*	90.54 ± 0.38 ^a^	16.49 ± 0.92 ^b^	5.09 ± 0.38 ^b^	22.90 ± 0.62 ^c^	23.51 ± 1.77 ^c^	36.04 ± 0.27 ^a^	54.68 ± 1.22 ^d^	1.49 ± 0.06 ^d^	0.63 ± 0.03 ^b^
**SEM**	0.177	0.532	0.201	0.553	0.766	0.355	0.883	0.037	0.016
***p*** **-value**									
LAB inoculation	0.007	<0.001	0.008	<0.001	0.115	<0.001	<0.001	0.021	0.432
Extract source	<0.001	<0.001	<0.001	<0.001	<0.001	<0.001	<0.001	<0.001	<0.001
Forage type	<0.001	<0.001	<0.001	<0.001	<0.001	<0.001	<0.001	<0.001	<0.001
LAB × Extract	0.026	<0.001	<0.001	<0.001	<0.001	0.009	<0.001	0.001	<0.001
LAB × Forage	<0.001	<0.001	<0.001	<0.001	<0.001	<0.001	<0.001	<0.001	<0.001
Extract × Forage	<0.001	<0.001	<0.001	<0.001	<0.001	<0.001	<0.001	<0.001	<0.001
LAB × Extract × Forage	<0.001	<0.001	<0.001	<0.001	<0.001	<0.001	<0.001	<0.001	<0.001

Different superscript letters in the same column indicate significant effect (*p* < 0.05). SEM = standard error of the mean. LAB = lactic acid bacteria. DM = dry matter; CP = crude protein; EE = ether extract; CF = crude fiber; NDF = neutral detergent fiber; ADF = acid detergent fiber; Ca = calcium; P = phosphorus. *p*-values indicate the significance of the main effects of LAB inoculation, extract source, forage type, and their two- and three-way interactions.

**Table 7:** Effect of phytobiotic and probiotic use on essential amino acid content.

Treatment Factors	Histidine (%)	Isoleucine (%)	Leucine (%)	Lysine (%)	Methionine (%)	Phenylalanine (%)	Threonine (%)	Tryptophan (%)	Valine (%)
**LAB inoculation treatments**									
Non-LAB	1.01 ± 0.05 ^a^	1.03 ± 0.05 ^a^	2.92 ± 0.09	2.58 ± 0.06 ^b^	0.75 ± 0.02	1.97 ± 0.06 ^b^	1.96 ± 0.06 ^a^	0.36 ± 0.01 ^a^	1.53 ± 0.05 ^a^
LAB	1.05 ± 0.05 ^b^	1.09 ± 0.06 ^b^	2.94 ± 0.09	2.39 ± 0.04 ^a^	0.76 ± 0.03	1.92 ± 0.07 ^a^	2.03 ± 0.06 ^b^	0.37 ± 0.01 ^b^	1.60 ± 0.05 ^b^
**Extract source treatments**									
Non-Extract	0.97 ± 0.05 ^a^	0.71 ± 0.06 ^a^	1.91 ± 0.10 ^a^	2.39 ± 0.07 ^a^	0.59 ± 0.03 ^a^	1.15 ± 0.05 ^a^	1.50 ± 0.05 ^a^	0.43 ± 0.02 ^c^	1.14 ± 0.07 ^a^
Acacia extract	1.10 ± 0.07 ^c^	1.24 ± 0.06 ^c^	3.18 ± 0.11 ^c^	2.60 ± 0.07 ^c^	0.83 ± 0.03 ^c^	2.18 ± 0.08 ^c^	2.02 ± 0.06 ^b^	0.42 ± 0.01 ^c^	2.09 ± 0.06 ^d^
Mahogany extract	1.05 ± 0.07 ^bc^	0.87 ± 0.08 ^b^	2.65 ± 0.10 ^b^	2.49 ± 0.06 ^b^	0.63 ± 0.03 ^b^	1.79 ± 0.07 ^b^	2.92 ± 0.05 ^c^	0.35 ± 0.02 ^b^	1.39 ± 0.06 ^b^
Cumin essential oil	1.01 ± 0.07 ^ab^	1.42 ± 0.06 ^d^	4.00 ± 0.08 ^d^	2.42 ± 0.09 ^ab^	0.97 ± 0.02 ^d^	2.64 ± 0.05 ^d^	1.52 ± 0.05 ^a^	0.26 ± 0.02 ^a^	1.61 ± 0.05 ^c^
**Forage type treatments**									
*Pennisetum purpureum*	0.30 ± 0.03 ^a^	0.57 ± 0.04 ^b^	2.72 ± 0.13 ^b^	1.74 ± 0.03 ^a^	0.72 ± 0.03 ^b^	2.13 ± 0.09 ^c^	1.51 ± 0.07 ^a^	0.22 ± 0.01 ^a^	1.04 ± 0.05 ^a^
*Indigofera zollingeriana*	1.75 ± 0.02 ^d^	1.39 ± 0.03 ^c^	3.37 ± 0.09 ^c^	3.08 ± 0.04 ^d^	0.93 ± 0.02 ^c^	2.00 ± 0.06 ^b^	2.11 ± 0.06 ^c^	0.51 ± 0.01 ^d^	1.92 ± 0.04 ^c^
*Gliricidia sepium*	1.20 ± 0.03 ^c^	1.75 ± 0.03 ^d^	3.44 ± 0.03 ^c^	2.63 ± 0.05 ^c^	0.92 ± 0.02 ^c^	2.28 ± 0.06 ^d^	2.33 ± 0.06 ^d^	0.44 ± 0.01 ^c^	2.01 ± 0.04 ^d^
*Stylosanthes guianensis*	0.86 ± 0.02 ^b^	0.54 ± 0.06 ^a^	2.21 ± 0.06 ^a^	2.47 ± 0.04 ^b^	0.46 ± 0.03 ^a^	1.37 ± 0.10 ^a^	2.01 ± 0.11 ^b^	0.28 ± 0.01 ^b^	1.26 ± 0.06 ^b^
**SEM**	0.033	0.037	0.065	0.035	0.017	0.044	0.043	0.008	0.035
***p*** **-value**									
LAB inoculation	0.016	<0.001	0.464	<0.001	0.389	0.039	<0.001	0.001	<0.001
Extract source	<0.001	<0.001	<0.001	<0.001	<0.001	<0.001	<0.001	<0.001	<0.001
Forage type	<0.001	<0.001	<0.001	<0.001	<0.001	<0.001	<0.001	<0.001	<0.001
LAB × Extract	<0.001	<0.001	<0.001	<0.001	<0.001	<0.001	<0.001	0.021	<0.001
LAB × Forage	0.294	<0.001	<0.001	<0.001	<0.001	<0.001	<0.001	0.224	<0.001
Extract × Forage	<0.001	<0.001	<0.001	<0.001	<0.001	<0.001	<0.001	<0.001	<0.001
LAB × Extract × Forage	<0.001	<0.001	<0.001	<0.001	<0.001	<0.001	<0.001	<0.001	<0.001

Different superscript letters in the same column indicate significant effect (*p* < 0.05). SEM = standard error of the mean. LAB = lactic acid bacteria. *p*-values indicate the significance of the main effects of LAB inoculation, extract source, forage type, and their two- and three-way interactions.

**Table 8:** Effect of the use of phytobiotics and probiotics on non-essential amino acid content.

Treatment Factors	Alanine (%)	Arginine (%)	Aspartic Acid (%)	Cystine (%)	Glycine (%)	Glutamic Acid (%)	Proline (%)	Serine (%)	Tyrosine (%)
**LAB inoculation treatments**									
Non-LAB	1.54 ± 0.05 ^b^	0.84 ± 0.04 ^b^	3.35 ± 0.18	0.49 ± 0.02 ^a^	1.24 ± 0.03 ^a^	1.24 ± 0.08 ^a^	0.97 ± 0.05	1.39 ± 0.03 ^a^	1.98 ± 0.05
LAB	1.45 ± 0.05 ^a^	0.77 ± 0.03 ^a^	3.32 ± 0.08	0.52 ± 0.02 ^b^	1.29 ± 0.03 ^b^	1.48 ± 0.10 ^b^	1.00 ± 0.07	1.41 ± 0.03 ^b^	2.01 ± 0.06
**Extract source treatments**									
Non-Extract	1.45 ± 0.10 ^ab^	0.61 ± 0.04 ^a^	3.14 ± 0.34 ^a^	0.40 ± 0.02 ^a^	1.20 ± 0.04 ^a^	1.17 ± 0.11 ^b^	0.41 ± 0.02 ^a^	1.16 ± 0.03 ^a^	1.32 ± 0.04 ^a^
Acacia extract	1.60 ± 0.06 ^c^	0.88 ± 0.07 ^c^	2.93 ± 0.09 ^a^	0.51 ± 0.02 ^b^	1.36 ± 0.02 ^c^	2.45 ± 0.15 ^c^	1.07 ± 0.07 ^c^	1.38 ± 0.03 ^c^	2.37 ± 0.06 ^c^
Mahogany extract	1.43 ± 0.06 ^a^	0.89 ± 0.04 ^c^	4.05 ± 0.09 ^b^	0.52 ± 0.02 ^b^	1.33 ± 0.04 ^b^	0.96 ± 0.08 ^a^	0.61 ± 0.05 ^b^	1.28 ± 0.03 ^b^	1.95 ± 0.06 ^b^
Cumin essential oil	1.49 ± 0.07 ^b^	0.83 ± 0.04 ^b^	3.22 ± 0.11 ^a^	0.58 ± 0.02 ^c^	1.18 ± 0.05 ^a^	0.86 ± 0.08 ^a^	1.84 ± 0.07 ^d^	1.81 ± 0.03 ^d^	2.34 ± 0.08 ^c^
**Forage type treatments**									
*Pennisetum purpureum*	0.91 ± 0.03 ^a^	0.32 ± 0.01 ^a^	2.10 ± 0.05 ^a^	0.27 ± 0.01 ^a^	0.97 ± 0.03 ^a^	1.16 ± 0.10 ^a^	1.29 ± 0.09 ^d^	1.17 ± 0.03 ^a^	2.24 ± 0.09 ^c^
*Indigofera zollingeriana*	1.26 ± 0.06 ^d^	0.99 ± 0.04 ^c^	3.71 ± 0.06 ^b^	0.73 ± 0.01 ^d^	1.58 ± 0.02 ^d^	1.33 ± 0.12 ^b^	1.03 ± 0.11 ^c^	1.52 ± 0.04 ^c^	1.99 ± 0.06 ^b^
*Gliricidia sepium*	1.63 ± 0.03 ^c^	0.90 ± 0.04 ^b^	3.89 ± 0.08 ^b^	0.56 ± 0.01 ^c^	1.49 ± 0.02 ^c^	1.61 ± 0.18 ^c^	0.75 ± 0.07 ^a^	1.66 ± 0.03 ^d^	2.21 ± 0.06 ^c^
*Stylosanthes guianensis*	2.17 ± 0.06 ^b^	1.00 ± 0.05 ^c^	3.63 ± 0.33 ^b^	0.45 ± 0.01 ^b^	1.04 ± 0.03 ^b^	1.35 ± 0.09 ^b^	0.85 ± 0.04 ^b^	1.26 ± 0.04 ^b^	1.54 ± 0.09 ^a^
**SEM**	0.036	0.025	0.097	0.011	0.019	0.065	0.042	0.021	0.041
***p*** **-value**									
LAB inoculation	<0.001	<0.001	0.843	<0.001	<0.001	<0.001	0.147	0.002	0.324
Extract source	<0.001	<0.001	<0.001	<0.001	<0.001	<0.001	<0.001	<0.001	<0.001
Forage type	<0.001	<0.001	<0.001	<0.001	<0.001	<0.001	<0.001	<0.001	<0.001
LAB × Extract	<0.001	<0.001	0.309	<0.001	<0.001	<0.001	<0.001	<0.001	0.002
LAB × Forage	<0.001	<0.001	0.432	0.029	<0.001	<0.001	<0.001	<0.001	<0.001
Extract × Forage	<0.001	<0.001	0.081	0.001	<0.001	<0.001	<0.001	<0.001	<0.001
LAB × Extract × Forage	<0.001	<0.001	0.527	<0.001	<0.001	<0.001	<0.001	<0.001	<0.001

Different superscript letters in the same column indicate significant effect (*p* < 0.05). SEM = standard error of the mean. LAB = lactic acid bacteria. *p*-values indicate the significance of the main effects of LAB inoculation, extract source, forage type, and their two- and three-way interactions.

## Data Availability

The database used and analyzed is available from the corresponding authors upon reasonable request.

## Supplementary Materials

The following supporting information can be downloaded at: https://www.mdpi.com/article/10.3390/ani16142224/s1, Table S1. Calibration and validation statistics of the near-infrared spectroscopy (NIRS) models used for predicting nutrient and amino acid composition in feed samples (N = 320).
