# Supplementary material for: Combined Application of Probiotic and Phytobiotic Additives Improves Fermentation Quality and Amino Acid Preservation in Tropical Forage Silages
Source: Animals (Basel). 2026 Jul 17;16(14):2224. doi: 10.3390/ani16142224 (PMC13406032; doi:10.3390/ani16142224)
Supplement: Supplementary file 1 [file animals-16-02224-s001.zip › animals-4370876-supplementary.pdf]

Supplementary Materials

Combined Application of Probiotic and Phytobiotic Additives Improves Fermentation Quality and Amino Acid Preservation in Tropical Forage Silages

Supplementary Table S1. Calibration and validation statistics of the near-infrared spectroscopy (NIRS) models used for predicting nutrient and amino acid composition in feed samples (N = 320).

Parameters	N	Calibration range (%)	Action range (%)	Max. Residual	Method	SEP	Bias	Slope
**Nutrient content**								
Dry Matter	320	83.94 - 95.40	83.94 - 95.40	0.0136	PLS	0.4512	0	1
Crude protein	320	1.34 - 68.58	1.34 - 68.58	0.0158	PLS	0.7443	0	1
Ether extract	320	0.20 - 16.10	0.20 - 16.10	0.0135	PLS	0.4195	0	1
Crude fiber	320	0.54 - 32.31	0.54 - 32.31	0.0011	PLS	0.6548	0	1
Ash	320	1.20 - 36.70	1.20 - 36.70	0.0013	PLS	1.0167	0	1
Neutral detergent fiber	320	10.18 - 39.21	10.18 - 39.21	0.0064	PLS	0.4010	0	1
Acid detergent fiber	320	16.96 - 55.50	16.96 - 55.50	0.0003	PLS	0.8815	0	1
Calcium	320	0.13 - 1.82	0.13 - 1.82	0.0014	PLS	0.0871	0	1
Phosphorus	320	0.16 - 1.59	0.16 - 1.59	0.0066	PLS	0.0754	0	1
**Essential amino acid content**								
Hystidine	320	0.13 - 3.48	0.13 - 3.48	0.0065	PLS	0.1732	0	1
Isoleucine	320	0.04 - 4.81	0.04 - 4.81	0.0023	PLS	0.1075	0	1
Leucine	320	0.77 - 8.65	0.77 - 8.65	0.0049	PLS	0.1524	0	1
Lysine	320	0.05 - 7.89	0.05 - 7.89	0.0006	PLS	0.2279	0	1
Methionine	320	0.14 - 2.05	0.14 - 2.05	0.0008	PLS	0.0514	0	1
Phenilalanin	320	0.03 - 6.55	0.03 - 6.55	0.0022	PLS	0.1006	0	1
Threonin	320	0.03 - 5.34	0.03 - 5.34	0.0006	PLS	0.0892	0	1
Tryptophan	320	0.03 - 1.48	0.03 - 1.48	0.0042	PLS	0.0430	0	1
Valine	320	0.05 - 5.49	0.05 - 5.49	0.0011	PLS	0.1600	0	1
**Non-essential amino acid content**								
Alanine	320	0.03 - 4.58	0.03 - 4.58	0.0018	PLS	0.0704	0	1
Arginin	320	0.23 - 6.12	0.23 - 6.12	0.0146	PLS	0.2432	0	1
Aspartic	320	0.09 - 8.44	0.09 - 8.44	0.0080	PLS	0.3245	0	1
Cystine	320	0.05 - 2.84	0.05 - 2.84	0.0005	PLS	0.0835	0	1
Glycine	320	0.06 - 4.84	0.06 - 4.84	0.0000	PLS	0.1010	0	1

Glutamic	320	0.22 - 29.56	0.22 - 29.56	0.0054	PLS	0.3407	0	1
Proline	320	0.61 - 12.84	0.61 - 12.84	0.0067	PLS	0.2546	0	1
Serine	320	0.04 - 5.31	0.04 - 5.31	0.0122	PLS	0.1000	0	1
Tyrosine	320	0.17 - 4.99	0.17 - 4.99	0.0024	PLS	0.1115	0	1

PLS, partial least squares regression; SEP, standard error of prediction.

Note: N represents the number of samples included in dataset. The calibration range indicates the concentration interval of samples used to develop the calibration model, whereas the action range represents the recommended concentration interval for routine prediction. Calibration models were developed using partial least squares (PLS) regression. Maximum residual represents the largest prediction residual observed during model development. SEP denotes the standard error of prediction, Bias is the mean difference between predicted and reference values, and Slope is the regression slope between predicted and reference values, where values approaching 1 indicate good agreement between predicted and reference measurements. Predictions for samples with concentrations outside the action range should be interpreted with caution because model accuracy may decrease.
